# 原发与继发胰腺弥漫大B细胞淋巴瘤的临床特征及预后分析

**DOI:** 10.3760/cma.j.issn.0253-2727.2023.01.010

**Published:** 2023-01

**Authors:** 雨佳 霍, 慕晨 张, 晴 施, 维 秦, 子旸 石, 黎 王, 澍 程, 彭鹏 许, 维莅 赵

**Affiliations:** 上海交通大学医学院附属瑞金医院血液科，医学基因组学国家重点实验室，上海血液学研究所，上海 200025 State Key Laboratory of Medical Genomics, Shanghai Institute of Hematology, Shanghai Rui Jin Hospital, Shanghai Jiao Tong University School of Medicine, Shanghai 200025, China

**Keywords:** 胰腺, 淋巴瘤，大B细胞，弥漫性, 临床特征, 突变, 预后, Pancreas, Lymphoma, large B-cell, diffuse, Clinical features, Mutation, Prognosis

## Abstract

**目的:**

研究原发与继发胰腺弥漫大B细胞淋巴瘤（DLBCL）患者的临床特征及预后。

**方法:**

回顾性分析2003年4月至2020年6月就诊于上海交通大学医学院附属瑞金医院的胰腺DLBCL患者的临床资料，采用靶向测序（55个淋巴瘤相关基因）评估患者的基因突变情况，采用单因素和多因素Cox回归模型评估患者总生存（OS）和无进展生存（PFS）的影响因素。

**结果:**

共纳入80例患者，其中原发胰腺DLBCL 12例，继发胰腺DLBCL 68例。与原发胰腺DLBCL相比，继发胰腺DLBCL患者结外受累数目较多（*P*<0.001）、国际预后指数（IPI）评分较高（*P*＝0.013）。原发与继发胰腺DLBCL患者OS和PFS的差异均无统计学意义（*P*值分别为0.120和0.067）。多因素分析结果显示，IPI评分中高危/高危（*P*＝0.025）和双表达（DE）（*P*＝0.017）是影响胰腺DLBCL患者OS的独立不良预后因素；IPI评分中高危/高危（*P*＝0.021）是影响胰腺DLBCL患者PFS的独立不良预后因素。29例患者的靶向测序结果显示，PIM1、SGK1、BTG2、FAS、MYC和MYD88在胰腺DLBCL患者中的突变率大于20％，其中PIM1突变（OS：*P*＝0.006，PFS：*P*＝0.032）和MYD88突变（OS：*P*＝0.001，PFS：*P*＝0.017）与继发胰腺DLBCL患者较差的OS和PFS相关。

**结论:**

原发与继发胰腺DLBCL患者的生存无显著差异，IPI评分中高危/高危、DE是影响胰腺DLBCL患者预后的不良因素。PIM1、SGK1、BTG2、FAS、MYC和MYD88是胰腺DLBCL中常见的突变，且具有PIM1及MYD88突变的患者预后不佳。

## Abstract (en)

**Objective:**

To analyze the clinical characteristics and prognosis of primary and secondary pancreatic diffuse large B-cell lymphoma（DLBCL）.

**Methods:**

Clinical data of patients with pancreatic DLBCL admitted at Shanghai Rui Jin Hospital affiliated with Shanghai Jiao Tong University School of Medicine from April 2003 to June 2020 were analyzed. Gene mutation profiles were evaluated by targeted sequencing（55 lymphoma-related genes）. Univariate and multivariate Cox regression models were used to evaluate the prognostic factors of overall survival（OS）and progression-free survival（PFS）.

**Results:**

Overall, 80 patients were included; 12 patients had primary pancreatic DLBCL（PPDLBCL）, and 68 patients had secondary pancreatic DLBCL（SPDLBCL）. Compared with those with PPDLBCL, patients with SPDLBCL had a higher number of affected extranodal sites（*P*<0.001）and had higher IPI scores（*P*＝0.013）. There was no significant difference in the OS（*P*＝0.120）and PFS（*P*＝0.067）between the two groups. Multivariate analysis indicated that IPI intermediate-high/high risk（*P*＝0.025）and double expressor（DE）（*P*＝0.017）were independent adverse prognostic factors of OS in patients with pancreatic DLBCL. IPI intermediate-high/high risk（*P*＝0.021）was an independent adverse prognostic factor of PFS in patients with pancreatic DLBCL. Targeted sequencing of 29 patients showed that the mutation frequency of PIM1, SGK1, BTG2, FAS, MYC, and MYD88 in patients with pancreatic DLBCL were all >20％. PIM1（*P*＝0.006 for OS, *P*＝0.032 for PFS）and MYD88（*P*＝0.001 for OS, *P*＝0.017 for PFS）mutations were associated with poor OS and PFS in patients with SPDLBCL.

**Conclusion:**

There was no significant difference in the OS and PFS between patients with PPDLBCL and those with SPDLBCL. IPI intermediate-high/high risk and DE were adverse prognostic factors of pancreatic DLBCL. PIM1, SGK1, BTG2, FAS, MYC, and MYD88 were common mutations in pancreatic DLBCL. PIM1 and MYD88 mutations indicated worse prognosis.

弥漫大B细胞淋巴瘤（DLBCL）是具有高度异质性的侵袭性非霍奇金淋巴瘤，约三分之一的DLBCL起源于结外部位[1]。胰腺DLBCL在总体DLBCL中约占5％，分为原发胰腺DLBCL和继发胰腺DLBCL[2]。原发胰腺DLBCL起病于胰腺肿物，可有胰腺引流区域或远处的淋巴结累及，是最常见的原发胰腺淋巴瘤[3]–[4]。继发胰腺DLBCL较原发胰腺DLBCL常见，约三分之一的广泛淋巴结和结外累及非霍奇金淋巴瘤患者会继发累及胰腺[5]。本研究对本中心收治的80例胰腺DLBCL患者进行回顾性分析，旨在探究胰腺DLBCL患者的临床特征及预后。

## 病例与方法

1. 病例：收集2003年4月至2020年6月上海交通大学医学院附属瑞金医院收治的初治原发或继发胰腺DLBCL患者。完善血常规、生化常规、心电图、骨髓穿刺、全身CT或PET-CT等检查，所有患者均经淋巴结病理及免疫组织化学确诊，病理诊断标准参考世界卫生组织（WHO）2016年淋巴瘤分类标准[1]，临床按照Ann Arbor分期标准对患者进行分期，体能状态评分为美国东部肿瘤协作组（ECOG）评分，预后因素采用国际预后指数（IPI）。

2. 治疗方式：80例胰腺DLBCL患者中，23例接受了胰腺肿瘤切除术，全部接受了以利妥昔单抗、环磷酰胺、阿霉素、长春新碱和泼尼松（R-CHOP）方案为基础的治疗。

3. 疗效评估：按照2014年Lugano标准进行疗效评估[6]，具体分为完全缓解（CR）、部分缓解（PR）、疾病稳定（SD）、疾病进展（PD）。总有效率（ORR）为CR率与PR率之和。

4. 随访：采用电话、查阅患者住院病历及门诊随访记录的方式随访，随访截止日期为2021年8月31日，中位随访时间为24.33（0.37～195.63）个月。总生存（OS）期指自患者开始治疗至因任何原因死亡或随访截止的时间。无进展生存（PFS）期指自患者开始治疗至出现PD、复发、死亡或随访截止的时间。

5. 靶向测序检测基因突变：应用组织gDNA提取试剂盒（美国Promega公司产品）提取gDNA。取1 µg DNA制备DNA全基因组文库。使用PCR引物扩增目的基因（55个淋巴瘤相关基因，分别为ARID1A、ATM、B2M、BCL6、BTG1、BTG2、CARD11、CCND3、CD58、CD70、CD79A、CD79B、CIITA、CREBBP、DDX3X、DTX1、DUSP2、EBF1、EP300、EZH2、FAS、FBXW7、FOXO1、GNA13、HIST1H1C、HIST1H1E、IRF4、IRF8、KMT2C、KMT2D、LYN、MAPK7、MPEG1、MTOR、MYC、MYD88、NFKBIE、NOTCH1、NOTCH2、PIM1、PRDM1、PTPN6、SGK1、SOCS1、STAT3、STAT6、TBL1XR1、TET2、TMSB4X、TNFAIP3、TNFRSF14、TP53、TSC2、ZFP36L1、ZNF608），将目标区域DNA富集后，采用Novaseq（美国Illumina公司产品）测序平台进行测序。测序后原始数据通过CCDS、人基因组数据库（HG19）、dbSNP（v138）、1000 genomes、COSMIC、PolyPhen、SIFT等数据库进行生物信息学分析，确定致病基因突变位点。

6. 统计学处理：采用SPSS 22.0软件分析数据，定性资料用例数（百分比）表示，定量资料用中位数（范围）表示。分类数据采用卡方检验或Fisher精确检验分析，采用Kaplan-Meier法绘制生存曲线，应用Log-rank检验比较组间OS、PFS的差异。应用Log-rank检验进行单因素分析，*P*<0.1的因素纳入Cox比例风险回归模型进行多因素分析。*P*<0.05为差异有统计学意义。

## 结果

1. 临床特征分析：80例胰腺DLBCL患者的临床特征见表1，原发与继发胰腺DLBCL组结外受累数目（*P*<0.001）和IPI评分（*P*＝0.013）的差异有统计学意义。

**表1:** 原发和继发胰腺弥漫大B细胞淋巴瘤患者的临床特征比较［例（％）］

临床特征	原发组（12例）	继发组（68例）	*χ*^2^值	*P*值
性别			NA	0.206
女	7（58.3）	25（36.8）		
男	5（41.7）	43（63.2）		
年龄			0.485	0.486
≤60岁	8（66.7）	38（55.9）		
>60岁	4（33.3）	30（44.1）		
发病部位			6.866	0.199
全胰	2（16.7）	30（44.1）		
胰体和胰尾	4（33.3）	10（14.7）		
胰尾	2（16.7）	12（17.6）		
胰头	4（33.3）	10（14.7）		
胰体	0（0）	6（8.8）		
钩突部位	0（0）	1（1.5）		
是否有B症状			0.009	1.000
否	6（50.0）	35（51.5）		
是	6（50.0）	33（48.5）		
Ann Arbor分期			NA	0.219
Ⅰ～Ⅱ	2（16.7）	4（5.9）		
Ⅲ～Ⅳ	10（83.3）	64（94.1）		
LDH升高			NA	0.711
否	3（25.0）	14（20.6）		
是	9（75.0）	54（79.4）		
ECOG评分			NA	0.679
0～1	11（91.7）	56（82.8）		
>1	1（8.3）	12（17.2）		
结外受累数目			NA	<0.001
0～1	11（91.7）	3（4.4）		
≥2	1（8.3）	65（95.6）		
IPI评分			9.629	0.013
0～1	3（25.0）	3（4.4）		
2	3（25.0）	9（13.2）		
3	5（41.7）	26（38.2）		
4～5	1（8.3）	30（44.1）		
COO^a^			NA	0.261
GCB	1（10.0）	19（32.2）		
non-GCB	9（90.0）	40（67.8）		
是否为DE^b^			NA	0.292
是	6（66.7）	23（45.1）		
否	3（33.3）	28（54.9）		

注 LDH：乳酸脱氢酶；ECOG评分：美国东部肿瘤协作组体力状况评分；IPI：国际预后指数；COO：细胞起源；GCB：生发中心来源；non-GCB：非生发中心来源；DE：双表达；NA：不适用；^a^总例数为69例，其中原发组10例，继发组59例；^b^总例数为60例，其中原发组9例，继发组51例

原发胰腺DLBCL中4例（33.3％）患者行手术切除病灶，继发胰腺DLBCL中19例（27.9％）患者行手术切除病灶。所有行手术切除的患者均完全切除胰腺病灶，切缘阴性。与未行手术切除的继发胰腺DLBCL患者相比，行手术切除的继发胰腺DLBCL患者LDH较低（*P*<0.001），Ann Arbor分期较低（*P*＝0.001），IPI评分较低（*P*<0.001）（表2）。

**表2:** 是否行手术切除的继发胰腺弥漫大B细胞淋巴瘤患者临床特征比较［例（％）］

临床特征	未行手术切除组（49例）	行手术切除组（19例）	*χ*^2^值	*P*值
性别			0.323	0.570
女	17（34.7）	8（42.1）		
男	32（65.3）	11（57.9）		
年龄			1.321	0.250
≤60岁	26（53.1）	13（68.4）		
>60岁	23（46.9）	6（31.6）		
是否有B症状			0.014	0.905
否	25（51.0）	10（52.6）		
是	24（49.0）	9（47.4）		
Ann Arbor分期			NA	0.001
Ⅰ～Ⅱ	0（0）	4（21.1）		
Ⅲ～Ⅳ	49（100.0）	15（78.9）		
LDH升高			NA	<0.001
否	3（6.1）	11（57.9）		
是	46（93.9）	8（42.1）		
ECOG评分			NA	1.000
0～1	40（81.6）	16（84.2）		
>1	9（18.4）	3（15.8）		
结外受累数目			NA	0.831
0～1	2（4.1）	1（5.3）		
≥2	47（95.9）	18（94.7）		
IPI评分			27.110	<0.001
0～1	0（0）	3（15.8）		
2	1（2.0）	8（42.1）		
3	24（49.0）	2（10.5）		
4～5	24（49.0）	6（31.6）		

注 LDH：乳酸脱氢酶；ECOG评分：美国东部肿瘤协作组体力状况评分；IPI：国际预后指数；NA：不适用

2. 疗效分析：80例胰腺DLBCL患者均可评估疗效。48例（60.0％）获得CR，5例（6.3％）获得PR，ORR为66.3％。68例继发胰腺DLBCL患者中40例获得CR，3例获得PR，ORR为63.2％；12例原发胰腺DLBCL患者中8例获得CR，2例获得PR，ORR为83.3％。原发与继发胰腺DLBCL组CR率（*P*＝0.754）和ORR（*P*＝0.320）的差异无统计学意义。

3. 生存分析：胰腺DLBCL患者的中位随访时间为24.33（0.37～195.63）个月，5年OS率和PFS率分别为29.2％和22.5％，其中原发胰腺DLBCL的5年OS率和PFS率分别为60.0％和50.0％，继发胰腺DLBCL患者的5年OS率和PFS率分别为24.2％和17.6％，原发和继发胰腺DLBCL的OS（*P*＝0.120）及PFS（*P*＝0.067）差异无统计学意义。

4. 预后的单因素和多因素分析：单因素分析结果显示，IPI中高危/高危（*P*＝0.021）和双表达（DE）（*P*＝0.030）是影响胰腺DLBCL患者OS的不良因素；IPI中高危/高危（*P*＝0.005）是影响PFS的不良因素（表3）。多因素分析结果显示，IPI中高危/高危（*HR*＝10.000，95％*CI* 1.340～74.580，*P*＝0.025）和DE（*HR*＝3.040，95％*CI* 1.220～7.550，*P*＝0.017）是影响胰腺DLBCL患者OS的独立不良预后因素；IPI中高危/高危（*HR*＝11.910，95％*CI* 1.450～97.570，*P*＝0.021）是影响胰腺DLBCL患者PFS的独立不良预后因素。

**表3:** 影响胰腺弥漫大B细胞淋巴瘤患者预后的单因素分析

因素	总生存期		无进展生存期	
	*HR*（95% *CI*）	*P*值	*HR*（95% *CI*）	*P*值
性别（男，女）	1.900（0.835～4.310）	0.126	1.230（0.633～2.390）	0.541
类型（继发，原发）	1.870（0.646～5.390）	0.249	2.160（0.838～5.590）	0.110
B症状（有，无）	1.530（0.722～3.240）	0.268	1.350（0.706～2.580）	0.365
IPI评分（中高危/高危，低危/低中危）	10.600（1.430～77.800）	0.021	7.890（1.890～32.900）	0.005
是否手术（是，否）	0.482（0.183～1.270）	0.140	0.499（0.219～1.140）	0.098
COO（non-GCB，GCB）	0.770（0.318～1.870）	0.563	0.696（0.336～1.440）	0.329
是否为DE（是，否）	2.730（1.100～6.760）	0.030	1.670（0.802～3.490）	0.170

注 IPI：国际预后指数；COO：细胞起源；non-GCB：非生发中心来源；GCB：生发中心来源；DE：双表达

5. 突变分析：共29例胰腺DLBCL患者（3例原发，26例继发）进行了靶向测序。3例原发胰腺DLBCL患者共检测出15个基因发生了突变，其中2例患者有PIM1基因突变。26例继发胰腺DLBCL患者中有1例未检测出突变，其余25例患者共检测出49个基因发生了突变，其中突变频率>20％的突变基因为PIM1、SGK1、MYC、BTG2、FAS和MYD88（图1）。生存分析显示，PIM1突变（OS：*P*＝0.006，PFS：*P*＝0.032）和MYD88突变（OS：*P*＝0.001，PFS：*P*＝0.017）与继发胰腺DLBCL患者较低的OS率和PFS率相关（图2）。

**图1:**
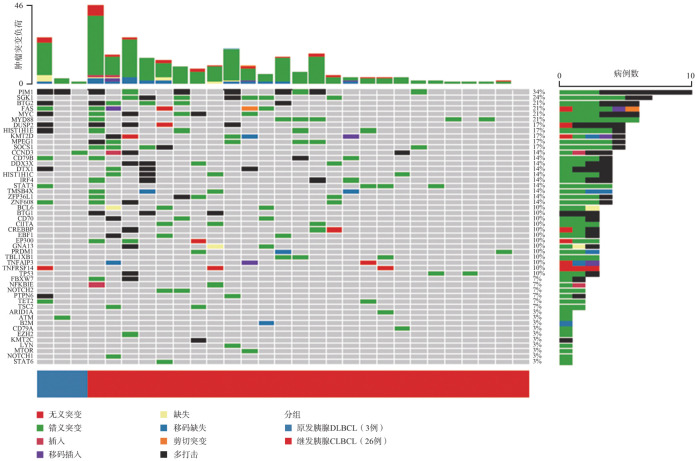
29例胰腺弥漫大B细胞淋巴瘤（DLBCL）患者的基因突变图谱

**图2:**
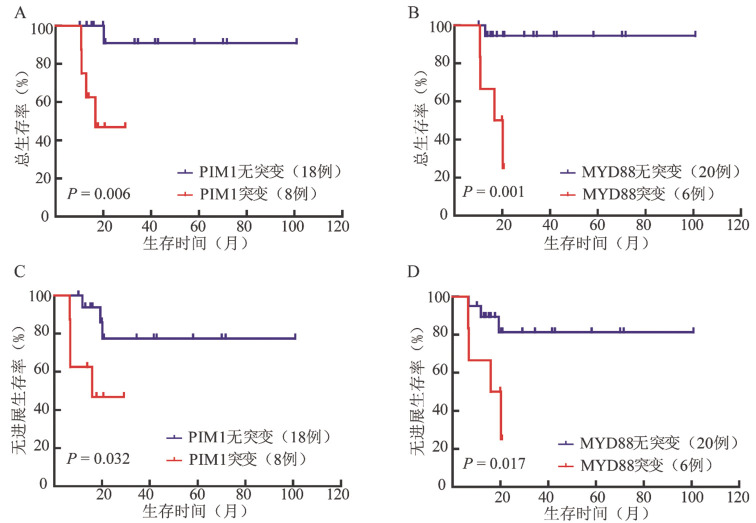
PIM1和MYD88基因突变对继发胰腺弥漫大B细胞淋巴瘤患者总生存（A、B）和无进展生存（C、D）的影响

## 讨论

胰腺DLBCL分为原发胰腺DLBCL和继发胰腺DLBCL，其中继发较原发常见。本研究结果提示，胰腺DLBCL多见于老年男性（60％），原发胰腺DLBCL最常见的胰腺累及部位为胰头（33.3％），其次为胰体和胰尾（33.3％），与文献报道相似[7]，继发胰腺DLBCL则通常累及全胰（42.6％）。此外，本研究中80例累及胰腺DLBCL患者中74例（92.5％）诊断时为晚期（Ⅲ～Ⅳ），其中原发胰腺DLBCL 10例（83.3％），继发胰腺DLBCL 64例（94.1％），表明累及胰腺DLBCL是一种恶性程度较高的淋巴瘤。原发胰腺DLBCL约占恶性胰腺肿瘤的0.5％[8]，其临床表现和影像学特征与胰腺癌相似，术前较难诊断[9]。有学者指出，原发胰腺DLBCL患者存在LDH增高，但胰腺癌患者LDH增高非常少见[10]。在本研究中，12例原发胰腺DLBCL中9例（75.0％）患者有LDH升高的表现，提示LDH升高对临床诊断原发性胰腺淋巴瘤有一定的参考价值。

本研究分析了胰腺DLBCL患者的病理特征。既往报道显示，DLBCL中non-GCB亚型占50％～60％，DE亚型占20％～35％[11]–[12]。本研究中，non-GCB亚型胰腺DLBCL患者占71.0％，DE亚型占48.3％，均较总体DLBCL高，可部分解释胰腺DLBCL分期较晚、预后较差的临床特点。

Shen等[2]分析了结外受累部位对DLBCL患者预后的影响，胰腺受累对PFS率降低有一定程度的影响，但差异不具有统计学意义（*P*＝0.065）。Castillo等[13]通过SEER数据库发现，原发肝/胰腺淋巴瘤患者的5年OS率低于50％，与呼吸道淋巴瘤相似，但较结内、头颈部和胃肠道淋巴瘤患者的OS率明显降低。本研究结果显示，原发胰腺DLBCL患者的5年OS率和PFS率分别为60.0％和50.0％，优于继发胰腺DLBCL患者的24.2％和17.6％，但差异无统计学意义，可进一步行大样本队列研究验证。与总体DLBCL患者5年OS率60％～70％相比[14]，胰腺DLBCL患者尤其是继发胰腺DLBCL患者的预后不佳，有待探索更强效的治疗方案。由于手术创伤大、无法彻底切除淋巴瘤、术后并发症较多等问题，手术是否有利于患者的生存始终存在争议[15]–[16]，有文献指出，对于术前无法确诊的原发胰腺淋巴瘤患者，可行手术明确诊断并减轻肿瘤负荷，随着胰腺手术水平和安全性的不断提高，会使部分患者获益[17]。本研究显示，是否手术与胰腺DLBCL患者预后的相关性不显著。此外，本研究通过多因素分析发现是否为DE和IPI评分可以判断胰腺DLBCL的预后，有助于早期识别预后不良的患者进行干预，对改善患者预后具有重要意义。

二代测序技术的出现及进步给疾病的诊治提供了新的思路。本探究首次应用靶向测序对29例胰腺DLBCL患者肿瘤组织样本中与淋巴瘤相关的55个基因的突变状态进行了检测。B细胞受体介导通路相关基因（MYD88、PIM1）、染色质重塑相关基因（SGK1）、细胞周期相关基因（BTG2、FAS、MYC）突变在胰腺DLBCL患者中较常见（突变频率>20％）[18]。进一步分析发现，PIM1和MYD88基因突变与患者的不良预后相关。PIM1是原癌基因，编码的蛋白质属于丝氨酸/苏氨酸蛋白激酶家族，其突变造成下游多条信号通路激活，进而促进肿瘤细胞生长增殖，是DLBCL患者常见的突变基因[19]。MYD88是Toll样受体信号通路中的一个关键接头分子，介导先天免疫，同时活化下游对B细胞生长有重要意义的NF-κB信号通路，其突变促进DLBCL肿瘤细胞的生长和分化[20]。Wright等[21]报道的DLBCL分子分型中，PIM1及MYD88突变属于MCD亚型，该亚型结外累及患者较多，且预后不良，与本研究结果一致。近年来，布鲁顿酪氨酸激酶（BTK）抑制剂联合化疗在DLBCL的治疗中显示出广阔的应用前景，在一项针对初治DLBCL患者的Ⅲ期临床试验（PHONIX）中，伊布替尼联合R-CHOP方案对non-GCB亚型患者的疗效显著优于R-CHOP方案，尤其是对于MCD和N1分子分型的患者[22]–[23]。故加用BTK抑制剂治疗胰腺DLBCL患者或许能够提高患者疗效。

综上，本研究提示胰腺DLBCL是一种恶性程度较高的非霍奇金淋巴瘤，预后较差，原发胰腺和继发胰腺DLBCL的生存无显著差异。IPI评分中高危/高危、DE、PIM1与MYD88突变是影响胰腺DLBCL患者预后的重要因素，BTK抑制剂和化疗联合使用或许能够改善胰腺DLBCL患者的预后。
